# An outbreak of *Mycoplasma pneumoniae* in children after the COVID-19 pandemic, Shanghai, China, 2023

**DOI:** 10.3389/fmicb.2024.1427702

**Published:** 2024-08-14

**Authors:** Xunhua Zhu, Pengcheng Liu, Hui Yu, Libo Wang, Huaqing Zhong, Menghua Xu, Lijuan Lu, Ran Jia, Liyun Su, Lingfeng Cao, Xiaowen Zhai, Yi Wang, Jin Xu

**Affiliations:** ^1^Department of Clinical Laboratory, National Children’s Medical Center, Children’s Hospital of Fudan University, Shanghai, China; ^2^Department of Infectious Diseases, Children’s Hospital of Fudan University, Shanghai, China; ^3^Department of Respiratory Medicine, Children’s Hospital of Fudan University, Shanghai, China; ^4^Department of Hematology/Oncology, Children's Hospital of Fudan University, Shanghai, China; ^5^Department of Neurology, Children’s Hospital of Fudan University, Shanghai, China; ^6^Shanghai Institute of Infectious Disease and Biosecurity, Fudan University, Shanghai, China

**Keywords:** *Mycoplasma pneumoniae*, COVID-19, P1 genotyping, macrolide resistance, non-pharmaceutical intervention, epidemiology

## Abstract

**Background:**

During the coronavirus disease 2019 (COVID-19) pandemic, the infection of *Mycoplasma pneumoniae* (MP) decreased significantly. At the beginning of the summer of 2023, there was an increasing trend of MP infection in China and the MP pneumonia (MPP) is surging when it comes to the school season and lasts for several months which has attracted widespread attention.

**Objective:**

This study aims to investigate the prevalent characteristics of the MP and the difference between the COVID-19 pandemic and the post in Shanghai, China.

**Methods:**

The demographic information and the results of laboratory pathogen detection from July 2021 to May 2024 were collected and analyzed to find out the prevalent characteristics of MP. Two periods, during the COVID-19 pandemic and the post-pandemic, were divided and compared. The P1 genotyping and macrolide resistance-associated gene of 23 s rRNA were detected using the remaining MP-positive samples.

**Results:**

During the COVID-19 pandemic, the prevalence of the MP has significantly decreased. Female children are more susceptible to MP infection than the male. The school-aged group (>6 years) had the highest infection rate. The rate of MP P1 genotype during post panel is higher than that during COVID-19 pandemic, which is dominant from July 2021 to May 2024, while the macrolide-resistant associated mutations (A2063G) keep high percentage during or post pandemic.

**Conclusion:**

After the COVID-19 pandemic, an outbreak of MP infection occurred from summer onwards in 2023 with children in Shanghai, China. Immunity debt and high rate of macrolide-resistance may take effects in this MP epidemic. Continuous surveillance of MP is necessary to help to alert the prevalence of MPP.

## 1 Introduction

*Mycoplasma pneumoniae* (*M. pneumoniae*, MP) was first isolated from the sputum sample of a patient having primary atypical pneumonia by Eaton et al. (1944). It is a crucial pathogen of community-acquired pneumonia (CAP) in children and is responsible for about 4%–8% of CAP cases in endemic periods and 40% in epidemics (Jain et al., 2015; Brown et al., 2016; Waites et al., 2017). It is transmitted by droplets from infected patients through the respiratory tract and can lead to respiratory infection, which is mild and self-limited generally but can still result in severe pneumonia and extrapulmonary manifestations particularly in the pandemic (Poddighe, 2020; Biagi et al., 2021).

MP is a kind of prokaryote without a rigid cell wall (Waites and Talkington, 2004). Thus, treatment of MP infection with beta-lactam antibiotics is ineffective. Macrolides, such as azithromycin, are recommended as the first-line antibiotics for treating children with MPP in many countries including China (Bradley et al., 2011; Tsai et al., 2021). However, with the wide application of macrolides, the resistance rate of MP to macrolides is increasing around the world, especially in China which is over 90% (Chen et al., 2020; Kim et al., 2022; Wang et al., 2022). The mechanism of macrolide resistance is the point mutations of the domain V in the 23 s ribosomal RNA with the frequent sites of A2063G/C/T, C2617G, and A2064G/C (Lucier et al., 1995). As for genotypes of MP, there are two major subtypes (type 1 and type 2) which aimed at the p1 adhesin gene.

During the epidemic of COVID-19, the prevalence of other respiratory pathogens like influenza A virus (IAV), respiratory syncytial virus (RSV), adenoviruses (ADV), and MP has significantly decreased (Liu et al., 2021; Chow et al., 2022; Ye and Liu, 2022; Meyer Sauteur and Beeton, 2023). The probable reason may be related to the adoption of strict non-pharmaceutical interventions (NPIs) like wearing masks, working at home, and closing schools, which blocked the spread of respiratory pathogens (Chow et al., 2022). Hence, experts predicted that an exceptionally large wave of MP infections could occur as a result of reduced exposure (Meyer Sauteur et al., 2022; Meyer Sauteur and Beeton, 2023). There have been reports of outbreaks of MP infection from Denmark, Netherlands, and France (Bolluyt et al., 2024; Nordholm et al., 2024; Zayet et al., 2024). Likewise, since the summer of 2023, outbreaks of MP pneumonia have emerged in various parts of China, characterized by the high rate of macrolide resistance and difficulty in the treatment of severe cases (Zhang et al., 2024).

Here, we conducted a retrospective epidemiologic analysis of MP between July 2021 and May 2024 spanning the COVID-19 pandemic (July 2021–December 2022) and the post (January 2023–May 2024) based on the “10 measures” policy announced by the China government at the onset of December 2022. The epidemiological characteristics, molecular type, and macrolide resistance were investigated in this study aiming to understand the MP prevalence better and reduce the morbidity and mortality caused by MP infection.

## 2 Methods

### 2.1 Study population

Patients with acute respiratory tract infection (ARTI) were enrolled at the Children’s Hospital of Fudan University in Shanghai, from July 2021 to May 2024. The respiratory specimens (bronchoalveolar lavage fluid/sputum/ nasopharyngeal swab) were collected by trained professional staff and then sent to the microbiology laboratory timely for a multi-pathogen detection using a commercial multiplex capillary electrophoresis PCR-based panel (Health Gene Technologies, Ningbo, China). It can detect 11 common respiratory pathogens simultaneously including MP, IAV, influenza B virus (IBV), human parainfluenza virus (HPIV), RSV, ADV, human metapneumovirus (HMPV), human rhinovirus (HRV), human bocavirus (HBOV), human coronavirus (HCOV), and chlamydia (CH). The medical records and the detection results were used to analyze the prevalence of MP and the remaining samples positive for MP were stored at −70°C for the subsequent macrolide-resistant mutations sequencing and P1 typing. Two periods, the COVID-19 pandemic and the post-pandemic, were set up. The patients were divided into five age groups: under 28 days of age (≤28 d), under 12 months of age (~12 m), 1–3 years of age (~3 y), 4–6 years of age (~6 y) and more than 6 years of age (>6 y).

### 2.2 Detection of macrolide resistance-associated mutations

The nucleic acid of the stored MP-positive samples was extracted and purified with a kit on an automated extraction machine from Tianlong, China. The nucleotide position 1,758–2,684 of the 23S rRNA gene was amplified by the primer pair prescribed previously (Matsuoka et al., 2004). Each amplification was performed on a T100 thermal cycler (Bio-Rad, United States) with a mixture of 25 μL containing 12.5 μL of TaKaRa premix Taq^TM^, 0.4 μM primers, 5 μL of template DNA, 5.5 μL of nuclease-free water. The reaction condition was as follows: initial pre-denaturation for 10 min at 95°C; followed by 40 cycles of 95°C for 30 s, 60°C for 30 s, and 72°C for 60 s; and a final extension at 72°C for 10 min. The amplification products were sequenced by Sanger sequencing (Sangon Biotech, Shanghai, China). All the sequences were aligned with the nucleotide sequence of *M. pneumoniae* M129 according to GenBank accession no. X68422 using MEGA11 software to seek out any point mutations.

### 2.3 P1 genotyping

A duplex real-time PCR assay described by Zhao et al. (2011) was used to identify the P1 genotypes of MP. The reaction mixture contained 12.5 μL of TaKaRa premix Ex TaqTM for probe, 0.5 μM of primers, 0.2 μM of probes, 5 μL of template DNA, and nuclease-free water to a final volume of 25 μL in total. The amplification conditions were as follows: 95°C for 2 min; followed by 45 cycles at 95°C for 15 s and 56°C for 15 s.

### 2.4 Statistical analysis

Continuous variables were presented as the mean or median, while the categorical variables were described by the number and percentage. Comparisons between different groups were conducted by Chi-square test using SPSS 23 (SPSS Inc., Chicago, IL, United States). A two-tailed *p*-value < 0.05 was considered statistically significant.

## 3 Results

### 3.1 Study population

After removing duplicate records, a total of 17,247 specimens of patients with ARTI were enrolled in this study, among which 4,461 (25.87%) were from the pandemic period and 12,786 (74.13%) from the post. The mean of the specimens during the pandemic was 248 cases per month but increased to 752 in the post-pandemic period due to the policy and prevalence alteration of COVID-19. The male was counted for 55.01% with a gender ratio of 1.22:1. The median age was 2 years old. The overall detection rate of MP was 27.29% (4,707/17,247). During the COVID-19 pandemic, the detection rate of MP was 8.34% (372/4,461) which was significantly lower than the post-pandemic period with a rate of 33.90% (4,335/12,786, *p* < 0.001).

### 3.2 Seasonality

**Figure 1:**
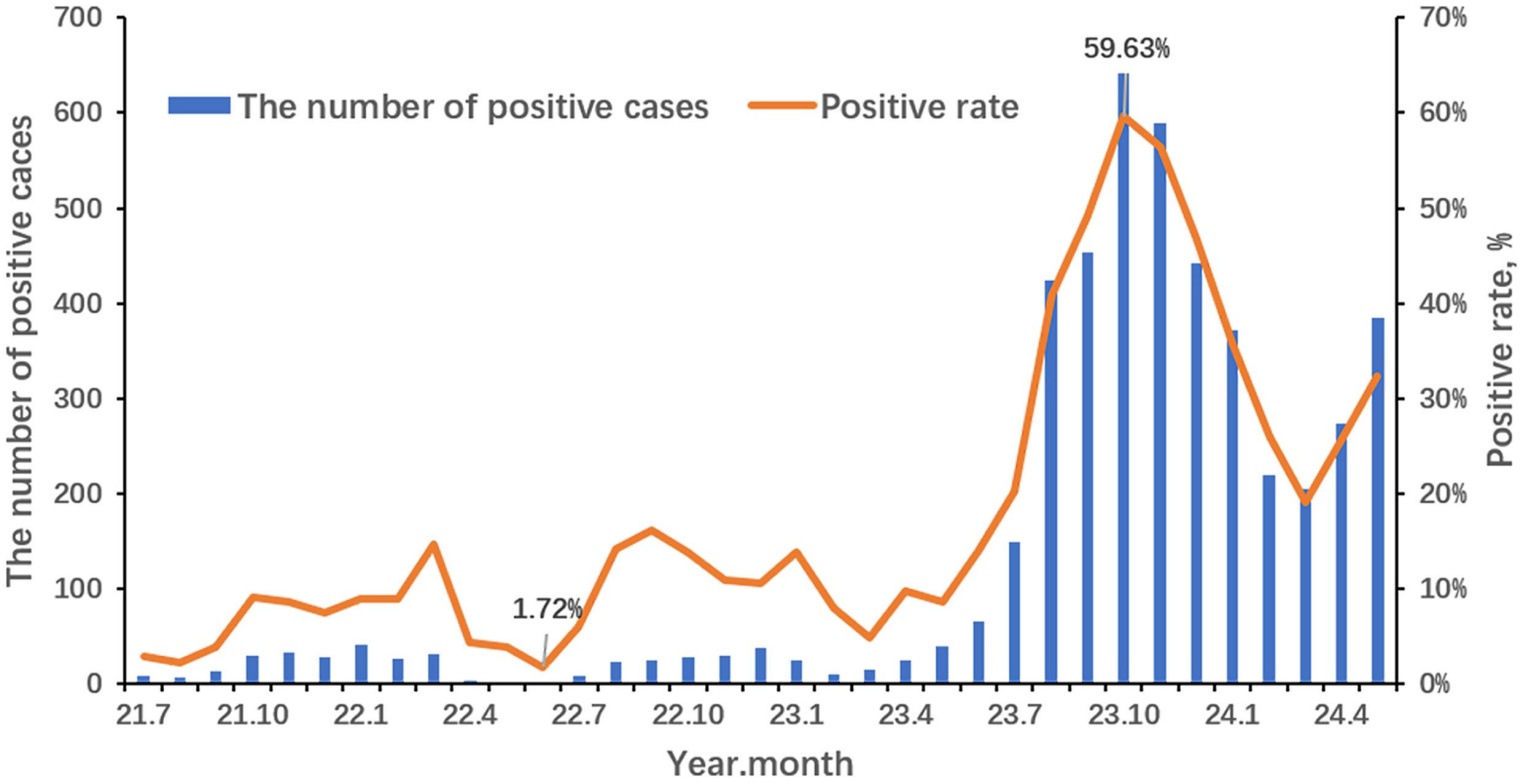
Seasonal distribution of MP from July 2021 to May 2024.

The seasonality profile and the epidemiological trends of MP infection from 2021 to 2024 are shown in Figure 1. The annual positive rates of MP from 2021 to 2024 were 5.91% (118/1,996), 10.30% (254/2,465), 38.03% (2,880/7,573) and 27.91% (1,455/5,213) respectively. During the COVID-19 pandemic, there is no obvious seasonality but a distinct reduction from April 2022 to June 2022. And the positive rate rises slowly from July 2022 to September 2022. It is suspected that there should be a small peak of MP in early summer to autumn but was disrupted by the lockdown policy in Shanghai stopping the spread of COVID-19. After the COVID-19 pandemic, the positive rate of MP increased relatively slowly from March to June 2023 and surged afterward. An outbreak has occurred since early summer, peaked in October, declined at a lowest level in March 2024, and risen again as the climate gets warmer. The positive rate of MP was the lowest in June 2022 (1.72%) and the highest in October 2023 (59.63%).

### 3.3 Age distribution of MP

**Figure 2:**
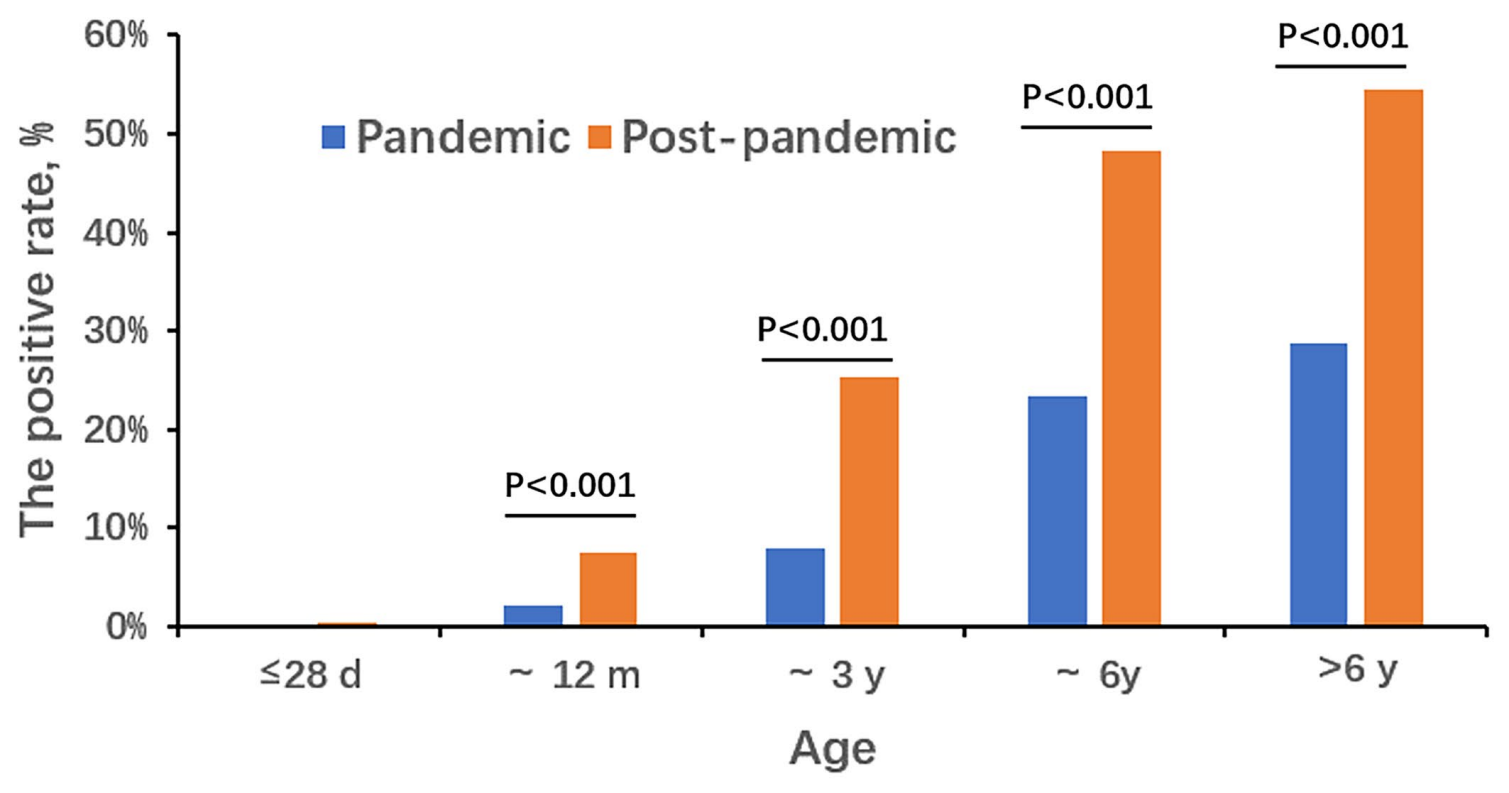
The positive rate of MP in five age groups during the pandemic and the post-pandemic.

During the COVID-19 pandemic, the detection rates for the five different age groups (≤ 28 d, ~12 m, ~3 y, ~6 y, and >6 y) were 0.00% (0/962), 2.25% (38/1,688), 7.85% (61/777), 23.43% (108/461), and 28.80% (165/573), respectively. After the pandemic, the rates were 0.42% (4/954), 7.44% (179/2,407), 25.27% (702/2,778), 48.29% (1,352/2,800), and 54.51% (2,097/3,847), respectively. Detection rates in newborns were the lowest, and school-age children over 6 years old were the highest. As age increased, the detection rate of MP gradually increased in two periods. Except the ≤28 d group, the positive rates of the post-pandemic period was higher than that of the pandemic in each age group (*p* < 0.001; Figure 2). The median age of MP-positive cases during the pandemic was 6 years old with the range of 0.15 to 17. In the post-pandemic period, the median age was 6 years old too but with the range of 0.02 to 17.

### 3.4 Gender distribution of MP

As shown in Table 1, the total detection rate of MP in female patients (30.30%) was higher than that in male patients (25.31%, *p* < 0.001). During and after the pandemic, the female proportions were both higher than the male patients, with statistical differences (*p* < 0.001) and the pandemic had no impact on the gender distribution of MP (Table 1).

**Table 1:** The gender distribution of MP.

Gender	the pandemic	Post-pandemic	Total
Male	177/2,510 (7.05%)	2,179/6,799 (32.05%)	2,356/9,309 (25.31%)
Female	195/1,951 (9.99%)	2,156/5,809 (37.11%)	2,351/7,760 (30.30%)
P	0.00	0.00	0.00

### 3.5 Co-infection

The co-infections in MP-infected patients were common and complex. Among the 4,707 positive samples from 2021 to 2024, a total of 1,972 samples were detected with multiple pathogens, accounting for 41.90% (1,972/4,707). The co-infection rate during the post-pandemic period (14.63%, 1,870/12,786) was higher than that during the pandemic (2.29%, 102/4,461, *p* < 0.001). Duplex infections were the most common accounting for 79.97%, with the highest incidence with HRV, followed by ADV, RSV, H3N2, and HCOV. Triple infections accounted for 17.19%, with the most common infection combinations being MP, HRV, and ADV. The co-infections with four and five pathogens accounted for 2.69% and 0.15%, respectively. The details of the co-infection situations are listed in Table 2.

**Table 2:** The co-infection types and proportions of MP.

Co-infection types	Number	Proportions
2 pathogens	1,577	79.97%
MP + HRV	624	31.64%
MP + ADV	343	17.39%
MP + RSV	114	5.78%
MP + H3N2a	93	4.72%
MP + HCOV	78	3.96%
MP + MPV	73	3.70%
MP + BOCA	49	2.48%
MP + INFB	39	1.98%
MP + INFB	6	0.46%
MP + CH	6	0.30%
MP + H1N1b	2	0.10%
3 pathogens	339	17.19%
MP + HRV + ADV	69	3.50%
MP + HRV + PIV	41	2.08%
MP + HRV + RSV	20	1.01%
MP + HRV + BOCA	20	1.01%
MP + ADV + PIV	16	0.81%
MP + HRV + MPV	15	0.76%
Others	158	8.01%
4 pathogens	53	2.69%
5 pathogens	3	0.15%

^a^Influenza virus of H3N2 subtype; ^b^Influenza virus of H1N1 subtype.

### 3.6 P1 genotypes

**Figure 3:**
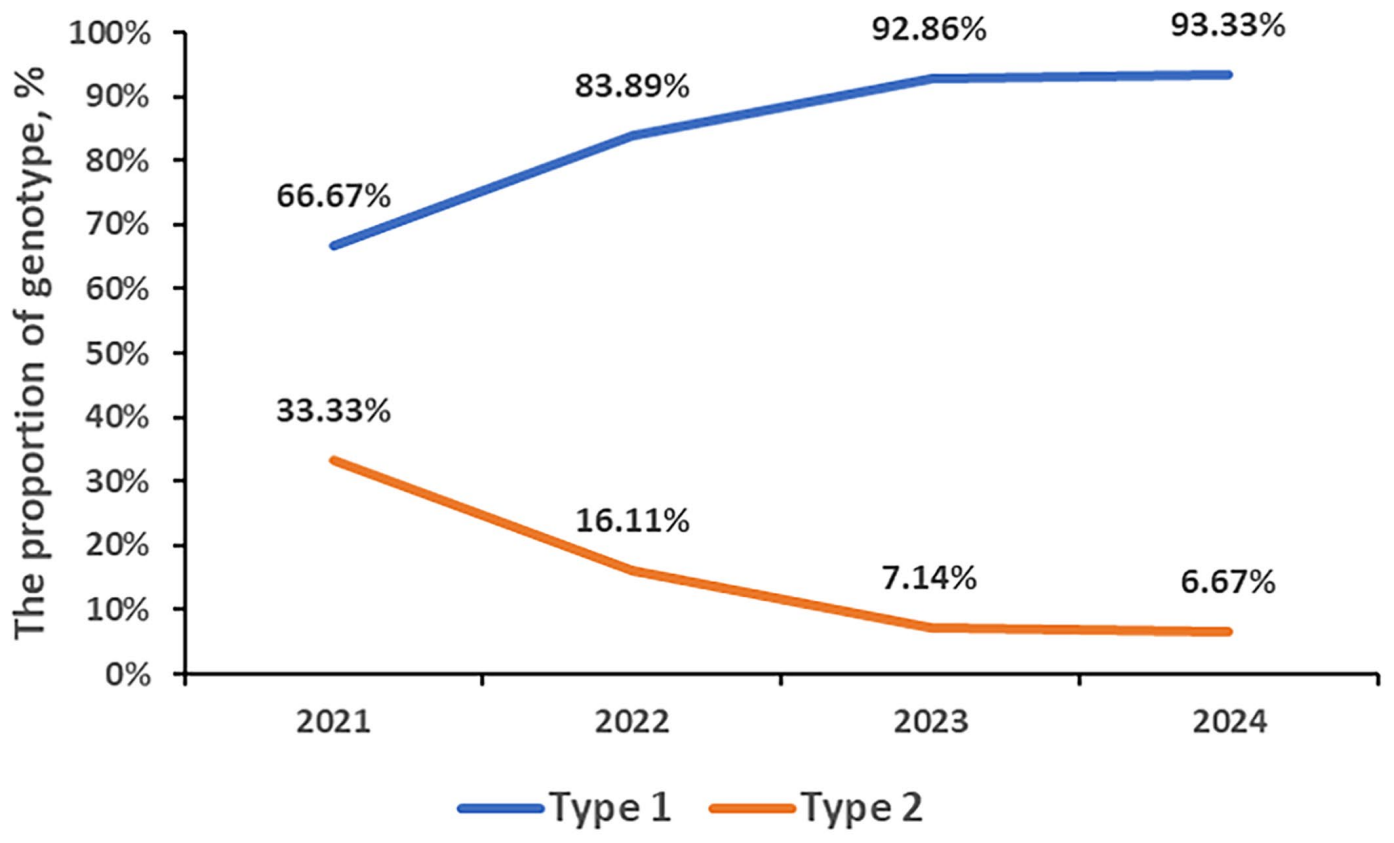
The prevalent trends of the P1 genotype.

A total of 835 samples were P1 genotyped. 731 samples (87.54%) were classified as type 1, and type 2 was identified in 104 cases (12.46%). From 2021 to 2024, the genotyped numbers were 111, 180, 364, and 180, respectively, and all of which were dominated by type 1. The proportion of type 1 has been increasing annually, reaching 66.67% in 2021, 83.89% in 2022, 92.86% in 2023, and 93.33% in 2024. The proportion of Type 2 has been decreasing yearly, reaching 33.33% in 2021, 16.11% in 2022, 7.14% in 2023, and 6.67% in 2024, respectively. The rate of MP P1 genotype during post panel (93.01%, 506/544) is higher than that during COVID-19 pandemic (77.32%, 225/291, *p* < 0.001; Figure 3).

### 3.7 Macrolide resistance-associated mutations

A total of 261 samples were sequenced successfully. A point mutation of A to G at position 2063 in domain V of the 23S rRNA gene was identified in 251 (96.17%) clinical samples. And no other mutations were found in all samples. From 2021 to 2024, 16, 78, 134, and 33 cases were successfully sequenced, with a resistance rate of 95.74% (90/94) during the pandemic and 96.41% (161/167) in the post. There was no significant difference in the macrolide-resistant associated mutations between the two groups (*p* = 1.000; Table 3).

**Table 3:** The sequencing results of macrolide resistance-associated mutations.

Mutation point	The pandemic		Post-pandemic		*P*
	2021	2022	2023	2024	
A2063G	16 (100%)	74 (94.87%)	128 (95.52)	33(100%)	
No mutation	0 (0%)	4 (5.13%)	6 (4.48%)	0 (0%)	
Total of A2063G	90(95.74%)		161(96.41%)		1.000

## 4 Discussion

Since COVID-19 was first discovered in December 2019 and spread all over the world quickly then, people got infected and affected by the large waves of outbreaks over the years and suffered a lot (Zhu et al., 2020). Though the clinical presentation caused by the Omicron strain is usually mild and at a low risk of hospitalization and death, long COVID and immune disorders have become more concerning issues (Klein et al., 2023). Meanwhile, the pandemic and the derived kinds of NPIs have had a huge impact on the epidemiology of other respiratory pathogens as well like influenza virus and RSV (Liu et al., 2021; Ye and Liu, 2022). In our study, the amounts of specimens for multiple-pathogen detection during the pandemic period are lower than the post as well as the MP-positive rate which means the pandemic influenced the circulation of MP negatively within the community.

It’s reported that the MP outbreaks occur every 3 to 7 years and the seasonal trend varies according to geography and temperature (Yan et al., 2016). In our study, the lowest monthly positive rate was in June 2022 right in the lockdown period in Shanghai. After that, the positive rate increased slowly until September 2022. It was suggested that there might be a small peak in the summer and autumn seasons in 2022 but was disrupted by the lockdown policy to stop COVID-19 from spreading. Since the “10 measures” policy was published in December 2022, kinds of NPIs were canceled. Respiratory pathogens start circulating in communities. At the onset of the summer of 2023, the MP-positive rates increased. Afterward, the MP outbreak occurred and swept through the children in Shanghai with the highest positive rate (59.63%) in October 2023 and still lasting till the spring of 2024. This means when an MP outbreak occurs, the prevalent period gets longer.

Consistent with many other studies (Kutty et al., 2019; Cheng et al., 2022), the most susceptible age group was in school-age children in our study both in the pandemic period and the post-pandemic. The reason may be that children in school, such a closed and concentrated place, are more likely to get infected by MP. It is worth noting that during the post-pandemic period, really young children even neonates get MP infection, while there were no cases in the pandemic period in the <28 d age group. This indicates that when an MP outbreak occurs, the susceptible age gets younger. As for the gender distribution, the positive rate of female patients is higher than that of males both in the two periods.

Previous studies showed that MP is likely to be co-infected with other respiratory pathogens, especially with viruses leading to a longer duration of fever, severe pneumonia, and poor response to the stepwise treatment of MPP (Gao et al., 2020; Choo et al., 2022; Li et al., 2022). In our study, the most common coinfected virus is HRV, which corresponds to the Soojeong Choo’s from Korea (Choo et al., 2022). ADV is another common and important virus co-infected with MP. According to the study of Shen Jun et al., children with severe CAP have high mixed detection rates of *Mycoplasma pneumoniae* and adenovirus in alveolar lavage fluid (ALF) samples (Li et al., 2022).

P1 genotyping, dividing MP into two subtypes of type 1 and type 2, is one of the most common methods of monitoring the molecular epidemiology of MP. A study from Japan showed that a type shift phenomenon occurs every 8–10 years and the type shift from one group to another requires 2–3 years (Kenri et al., 2008). In our study, the proportion of type 1 during 2021–2024 was 66.67%, 83.89%, 92.86%, and 93.33% respectively, which was increasing year by year. Correspondingly, the proportion of type 2 has been decreasing from 33.33% in 2021 to 6.67% in 2024. Thus, we predicted that the year 2021 to 2024 coincided with the period of type shift of MP and that type 1 will continue to dominate in the next few years.

Macrolides are used as the first-line antibiotics for treating MMP in children, China. Anne et al. reported an MP outbreak from October to December, in Denmark, detecting a low resistant proportion of less than 2% (Nordholm et al., 2024). However, in our study, the total macrolide-resistance rate over the 3 years was high to 96.17%. The extremely high drug resistance rate poses a serious challenge to the treatment of MPP. It’s vitally important to use macrolides reasonably and other treatments timely for MP-resistant patients.

There are several limitations in our study. Firstly, it’s a single-center and retrospective study, only radiating to Shanghai and its surrounding provinces and not representing the overall situation in China, though it is the largest children’s hospital in Shanghai. Secondly, due to the limited time of detecting nuclide acid of multiple respiratory pathogens, the study has a short period and needs further surveillance in the future.

In conclusion, we found that the macrolide-resistant mutations, the gender and age distribution did not have apparent changes. Immunity debt and high rate of macrolide-resistance may take responsibility for this MP epidemic and the large population mobility after the COVID-19 pandemic may take effects to the spread of MP. Continuous surveillance of MP and other respiratory pathogens is necessary to help to alert the prevalence of kinds of pathogens.

## Data availability statement

The original contributions presented in the study are included in the article/supplementary material, further inquiries can be directed to the corresponding authors.

## Ethics statement

The studies involving humans were approved by Children’s Hospital of Fudan University. The studies were conducted in accordance with the local legislation and institutional requirements. Written informed consent for participation was not required from the participants or the participants’ legal guardians/next of kin in accordance with the national legislation and institutional requirements.

## Author contributions

XuZ: Writing – original draft, Data curation, Investigation, Methodology, Resources, Software, Supervision, Visualization, Writing – review & editing. PL: Writing – review & editing, Methodology, Resources. HY: Writing – review & editing, Investigation, Resources. LW: Writing – review & editing, Investigation, Resources. HZ: Writing – review & editing, Data curation, Investigation, Methodology. MX: Writing – review & editing, Methodology, Resources, Software. LL: Writing – review & editing, Methodology, Project administration. RJ: Writing – review & editing, Investigation, Methodology, Resources, Software. LS: Writing – review & editing, Investigation, Resources. LC: Writing – review & editing, Data curation, Resources. XiZ: Writing – review & editing, Data curation, Resources. YW: Writing – review & editing, Data curation, Resources. JX: Writing – review & editing, Resources, Supervision, Validation.
